# Evaluating the higher education productivity of Chinese and European “elite” universities using a meta-frontier approach

**DOI:** 10.1007/s11192-021-03978-z

**Published:** 2021-04-26

**Authors:** Tommaso Agasisti, Guo-liang Yang, Yao-yao Song, Carolyn-Thi Thanh Dung Tran

**Affiliations:** 1 Politecnico di Milano School of Management, Milano, Italy; 2 Institutes of Science and Development, Chinese Academy of Sciences, Beijing, China; 3 Capital University of Economics and Business, Beijing, China; 4 NSW Public Policy Institute, The University of Sydney, Sydney, Australia

**Keywords:** Higher education productivity, Meta-frontier, Elite universities, I21, I23

## Abstract

This research focuses on a sample of European and Chinese elite universities for the period 2011–2015. We adopt a meta-frontier methodology to decompose their overall productivity in three main determinants: (1) technical efficiency compared with contemporaneous technology, (2) change in technical efficiency and (3) technology relative superiority of the two groups of universities. The results reveal different patterns of evolution: Chinese institutions’ productivity grows faster than that of their European counterparts (+ 7.15%/year vs 4.51%/year), however the latter maintain a higher level of technology in efficient production as a group.

## Introduction, motivation and research questions

In many areas of the world, policy-makers are defining intentional policies for creating and developing “world-class” and elite universities (Altbach & Salmi, 2011; Deem et al., 2008; Salmi, 2009; Shin, 2009a; Yang & Welch, 2012). While the specific literature about this field is far from reaching an overall consensus about a unique definition of what a world-class or elite university is (Huisman, 2008; Salmi 2009) identified three key elements that are now accepted by most scholars who work on this topic: (1) concentration of talents, (2) abundant resources and (3) favourable governance. These dimensions, albeit conceptually clear, are however difficult to be operationalized, and developing indicators for “measuring” the extent to which a university can be defined “elite” is actually very rare. A common shortcut, adopted by scholars and practitioners, is the use of rankings published by various authoritative entities, such as Quacquarelli Symonds (QS), Time Higher Education (THE), Financial Times, and the Shanghai Jiao Tong University’s Academic Ranking of World University (ARWU). Given the prestige associated with the number of elite universities in the country, many governments decided to launch specific initiatives for concentrating resources in some leading universities or for stimulating the growth of top-level academic institutions. Notable examples of such policies are the so-called “excellence initiatives” promoted in Germany (Kehm & Pasternack, 2009), Russia (Yudkevich et al., 2016), China (Zhang et al., 2013) and France (Boudard & Westerheijden, 2017).

In the context of such developments in the higher education (HE) sector, top-class universities in the various countries have strong incentives to continuously searching for improvements in their operations and results. Indeed, academic rankings place emphasis on performance indicators that measure the ability of reaching high levels of teaching, research and knowledge transfer (Buela-Casal et al., 2007; Johnes, 2018; Lukman et al., 2010). As a consequence, university managers decide how to allocate their resources to the most productive practices and initiatives such as, incentives for publications or the creation of attractive courses. Each institution starts regarding others like “competitor” in the same international higher education arena (Grewal et al., 2008). Globalization also is a force that is pressing universities in comparing themselves with potential competitors from all over the world (Stromquist, 2007).

This “battle” for elite and world-class universities (Hazelkorn, 2015), therefore, does not come without costs. Actually, institutions must invest a lot of human and financial resources to improve their performances in those dimensions which are valued by the agencies that compile rankings, such as the number of graduates, their employability, research outputs like grants, publications and citations. The innovative and high-quality activities that must be realized for pursuing these objectives are likely to generate unintended consequences, as for example an “excess of spending” in some activities which is not reflected in outputs’ volumes and quality. In this perspective, it would be useful to analyse the efficiency of elite universities, i.e. their ability to produce their high volumes of output at the minimum cost or, conversely, to maximize the output they produce with the available resources. Such a viewpoint related to the use of resources is a new one, which could go beyond the pure analysis of outputs activities and provide more economic insights about the way in which the elite institutions employ their resources.

In this paper, we aim at comparing the efficiency of a sample of Chinese and European elite universities. The rationale for choosing these two areas stem from the interest to develop and maintain groups of ‘elite’ universities competing in global rankings, which are still dominated by institutions in the USA (Aghion et al., 2010). The Chinese higher education system is recently concentrated on the world-class universities and first-class disciplines (Double-First Class) initiative construction, which aims to build a number of elite universities and disciplines until 2050 and further strengthen the quality of Chinese higher education. After the development of more than half a century, Europe has built up a world-class modern higher education system and many elite universities, which greatly promoted the rapid progress of science and technology worldwide. Comparing the efficiency of universities across countries and continents is an extraordinarily important task in this historical moment. For example, Wolszczak-Derlacz (2018) analyses how the productivity of US and European research universities evolved over time from 2000 to 2010, discovering that the groups experience a different pattern of efficiency (increasing in Europe, declining in the USA). The analysis presented in this paper conducts a similar, although innovative, exercise for a sample of elite universities in Europe and China for the period 2011–2015. Specifically, we answer the following questions: (1) Are Chinese elite universities more efficient than their European counterparts, or is the other way round? (2) How has the efficiency and productivity of European and Chinese elite universities evolved in the period under scrutiny? (3) Which are the main components of productivity that determine the relative efficiency of Chinese and European elite universities both within and across the two areas?

This paper innovates the current literature in three main ways. First, the research introduces the necessity of comparing elite universities not only on the ground of their performance (which is the traditional perspective) but also on the basis of their efficiency—i.e. the productivity rate at which they produce their level of output. Second, whilst comparative studies among different countries in Europe have been existing, a comparison of universities in Europe and China has yet been perused in the literature, thus this paper aims to represent the first attempt of comparing European and Chinese universities within the broader framework of the “battle” for world-class and elite status. Third, the comparison of the productivity of the selected universities is based on a meta-frontier approach, considering various components of performance—namely technical efficiency, intertemporal change and the comparison between the two groups of units.

The remainder of the paper is organized as follows. The next Sect. 2 contains a literature review about three main topics that influence our study: cross-country comparisons of universities’ efficiency, academic rankings and elite universities. Section 3 illustrates the methodological approach and describes the dataset we built for the empirical exercise. Section 4 reports the empirical results. Section 5 discusses the results, along with some concluding remarks.

## Literature review

Studies on education efficiency in a cross-nation comparative perspective has appealed much attention of researcher and scholar in recent years. These studies have been conducted in terms of the level of analysis, institutional or country, and the type of education, secondary schools or tertiary institutions (universities) that to some extent much depends on available large-scale databases (Aparicio et al., 2018).

For the purpose of cross-national assessment of the relative performance of tertiary education, some recent studies have concentrated on university efficiency and productivity across several nations, particularly in Europe. For example, Bonaccorsi et al. (2007) compared efficiency of institutions of higher education of six OECD nations while Wolszczak-Derlacz and Parteka (2011) conducted a study on performance of seven OECD countries. Using the two-stage Data Envelopment Analysis (DEA) in terms of output-orientated constant returns to scale approach, their findings showed that inefficiency of institutions could be attributed to some determinants such as scale economies, number of departments and funding. Instead comparing many countries in a common framework of analysis, Agasisti and Johnes (2009) looked into efficiency of HEIs in a pair of countries, Italy and the United Kingdom, using the Malmquist index based on DEA variable returns to scale method. The findings revealed that Italian universities are improving their technical efficiency scores whereas English institutions are obtaining stable scores during a period of 2002/03–2004/05. The authors argued that by comparison of only two nations, more qualitative information can be explored in terms of policies and that using a short panel data ensures any trends identified in data that are recent. Going with this stream, Agasisti and Perez-Esparrells (2010) analysed efficiency of Italian and Spanish State universities in a cross-country comparison using Malmquist productivity indexes based on the DEA variable returns to scale approach for the academic year 2004/05. They found that improvement in Italian HEIs was due to technological changes whereas the change in the performance of Spanish counterparts HEIs came from pure technical efficiency. Regional effects were also found to be determinants of inefficiency in these universities, respectively. In the same token, Agasisti and Wolszczak-Derlacz (2015) compared efficiency of universities in Italy and Poland using Malmquist productivity index based on the conventional DEA output-orientated and variable returns to scale method for a long panel data of 2001–2011. The authors used the bootstrap truncated regression procedure of Simar and Wilson (2007) to test the influence of external factors against the estimated DEA scores for each sample of Italy and Poland. Their results indicated that there was a great difference in performance of universities in two nations and that efficiency frontier has been more improved in Italy than Poland. Most recently, Agasisti and Gralka (2019) investigated the transient and persistent efficiency of 70 Italian and 76 German universities using a stochastic frontier analysis (SFA) for the period of 2001–2011. The findings showed that Italian universities are more efficient than their German counterparts when capturing persistent efficiency of universities in two nations over a long period.

As can be observed, the above studies comparing universities between two or more nations undertook estimates of efficiency in a specific-country frontier, meaning that they measured efficiency of universities separately and compared them based on shared characteristics as described in the beginning of their study. Alternatively, the framework is based on assuming a common international frontier, without considering any structural difference between the two different higher education systems. One might ask whether frontier technological gap between two or multiple groups of universities coming from two or several countries can be compared when all of them are put into a general meta-frontier framework. That is indeed an interesting question that contributes a novelty to the sector by seeking to use different advanced methods recently developed in the literature. This provides us an avenue to explore this breakthrough by relaxing the assumption of a common technology of production across institutions of interest—something that we pursue in this paper.

Whilst comparative studies across countries on efficiency and productivity of universities have been well noted in Europe, this is not the case in China whereby numerous studies on efficiency of Chinese universities have focused on only China without a comparison with other countries. Apart from this, such studies have used similar methods of analysis as in comparative studies of efficiency in European universities such as DEA, network DEA, Malmquist productivity index, and SFA to analyse the performance of universities in China—see, for example: Johnes and Li (2008), Ng and Li (2009), Yaisawarng and Ng (2014), Hu et al. (2017), Yang et al. (2018), Wu et al. (2020). This would lead us to a question whether a comparative study should be done between European and Chinese universities to investigate whether any gap in their performance exists, given that China’s national reforms and investment initiatives in higher education aiming at raising the research standards of key universities for the 21st Century as “*elite universities*” (Zong & Zhang, 2019).

The academic literature already debated what an elite university is, and how they can be defined and characterized. *Elite universities* are identified as “the presence of highly qualified faculty, talented students, abundant resources, autonomy, and favourable governance” (Abramo & D’Angelo, 2014, p. 1271). By the same token, Palfreyman and Tapper (2009) assert that the elite university is academically excellent with respect to educational qualification, teaching and research. However, elite universities are not necessarily world-class but could potentially lead to a world-class university in terms of their competitive advantage in quality of education and research resulting in the social and market value (Abramo & D’Angelo, 2014). The growth of elite universities should go with favorable policy environment and university autonomy in a nation’s context (Aghion et al., 2010). Otherwise, elite institutions could be inevitably absent in that nation, thus would lead to a brain drain phenomenon due to lack of high-qualified staff and students towards nations with world-class universities (Auranen & Nieminen, 2010; Abramo & D’Angelo, 2014).

Investment and development of domestically elite universities are considered as a pathway to reach the world-class universities via international ranking systems. The efforts to become world-class are absolutely helpful to enhancing academic outputs of research projects and publications (Altbach, 2004; Shin, 2009a; Song, 2018). The international ranking system and “elite” or “world-class” institutions are identified as a guideline for nation’s government and universities to form appropriate higher education policies and strategies for the purpose of moving university performance forward. Deem et al. (2008) presented that European universities have taken league tables such as the the Time World University Ranking (THE), the Shanghai Jiao Tong University’s Academic Ranking of World University (ARWU) as a central target of their reform process and thus suggested to increase higher education GDP investment by more than 2% per annum in future. Together with this, the Bologna process that aimed to achieve some degree of harmonization of higher education across European countries has been criticized because it does not fully address wider matters of globalization and internationalization (Kwiek, 2004). On top of this, Kwiek (2005) argued that the Bologna and other European Union higher education reform may be less successful because of the lack of funding of public universities and the varied status of universities with respect to other public services. International ranking, such as THE, ARWU, QS World University Ranking and Webometrics Ranking, has influenced higher education in Asian nations. For instance, in Hong Kong, university leaders are concerned about their institutions’ ranking in international ranking league whereas academic staff face challenges to publish their work in high ranked journals (Chan, 2007; Deem et al., 2008). Similarly, international publication venues are concerned by academics in Taiwan in terms of promotion and research evaluations (Chen & Lo, 2007). In Japan, after benchmarking of the world university ranking has been launched, the government has invested additional resources to promote internationalisation in terms of international collaboration and exchanges (Furushiro, 2006; Yonezawa, 2006). By the same token, the Chinese government has implemented some projects of higher education to enhance the internationalisation competitiveness of Chinese universities (Deem et al., 2008). For example, the greatest of these is the project 211 started in 1995. This project aims to enhance research standards in China’s elite universities and therefore receive considerably increased funding. In 2018, there are 116 institutions eligibly listed in the project 211 (Kumar & Thakur, 2019). These projects aim to enable Chinese institutions as national elite universities becoming world-class universities in the future. The second project was established in 2015 to develop 42 Chinese universities into the world-class universities by 2050. In addition, 39 universities are listed as more selective group, so-called Project 985. Finally, as part of Project 985, nine universities are formalized into China’s C9 League to foster better students and share resources (Deem et al., 2008; Kumar & Thakur, 2019).

In the contemporaneous dynamic world, albeit the international ranking systems could be controversial, they are widely accepted as benchmarking for universities around the world to perceive themselves as well as to be perceived by others. The government of various nations started to consider the international ranking as a guideline to form higher education policies and strategies for their nation’s universities to be ranked in the top list. However, what is the performance of a nation’s elite universities on the way reaching the world-class ranking based on their available input resources and academic outputs has been thoroughly debated only scantly; this is a gap in knowledge that should be explored to assist elite institutions to obtain the best practice in providing their education and training quality.

## Methodology and data

### Statistical methods employed for the empirical analyses

Traditional performance evaluation techniques commonly assume that decision-making units (DMUs, that is elite universities in our context) are situated in a homogeneous environment, so that they determine a single production function or reference set to identify the performance of different units accordingly. However, in the real scenario, the evaluated DMUs usually vary by individual characteristics and the environment, and heterogeneity characterizes the landscape of evaluated organizations. For instance, universities in different regions, countries or continents are faced with different evaluation and incentive mechanisms, verified funds and personnel allocation modes and other different features of the economic and social environment. This leads to a drawback in traditional approaches that they may lose their practicability when the evaluated DMUs are not homogeneous. Referencing the idea of constructing separate production frontiers and a common meta-frontier for different groups of individuals in Battese and Rao (2002), this paper uses a non-parametric meta-frontier technique (O’Donnell et al., 2008; Oh & Lee, 2010) and incorporates the Malmquist productivity index (Malmquist, 1953; Caves et al., 1982; Fare et al., 1994) to investigate the different performance of Chinese and European universities. Sickles and Zelenyuk (2019, p.98) reveal that “the concept of efficiency and the concept of productivity are different concepts that can explain the same thing, *performance*”. Following this, we used both terms, productivity and efficiency that refer to the performance of universities, given that the concepts of productivity variation can arise from different sources such as attributed to differences in production technology, differences in the scale of operation, differences in operating efficiency, and differences in the operating environment in which production occurs (Fried et al., 2008; Coelli et al., 2005). Thus, to make inferences about efficiency, the effect on productivity caused by the differences in operating environment and other exogenous factor should be removed (Oum et al., 1999).

Assume there are n DMUs to be evaluated, and each ${\text{DMU}}_{j} \left( {j = 1,2, \ldots ,n} \right)$ generates $S\left( {s = 1,2, \ldots ,S} \right)$ outputs using $M\left( {m = 1,2, \ldots ,M} \right)$ inputs in period $t\left( {t = 1,2, \ldots ,T} \right)$. The observation can be denoted as $\left( {x^{t} ,y^{t} } \right) \in R_{m}^{ + } \times R_{s}^{ + }$. We further suppose that there are $k = 1,2, \ldots ,K$ separated subgroups within the whole observations, and members inside each subgroup share the identical technology. In this paper, we assume that separate groups are European vs Chinese universities.

In the framework of meta-frontier analysis, three types of fundamental technology sets or production possibility sets can be introduced, namely (1) contemporaneous technology set, (2) intertemporal technology set and (3) global technology set.

The boundary (or frontier) of a *contemporaneous technology set* is composed of a group of best-performing DMUs, which are represented as the benchmark among DMUs within the same group during the same period. In practice, it allows estimating the most efficient universities with each of the two groups (European vs Chinese). Specifically, the contemporaneous technology set is defined as a reference set for all observations within group *k* in time *t*, and it is denoted as:

1 $$T_{k}^{t} = \left\{ {\left( {x^{t} ,y^{t} } \right)|x^{t} {\text{can produce}}\; y^{t} } \right\}, t = 1,2, \ldots ,T$$

Hence, observations in the same group *k* are easily classified into *T* contemporaneous technology sets according to different periods. Following the measurement of technical efficiency in Farrell (1957) and the formulation of output distance function in Shephard (1970), the efficiency of an observation inside the contemporaneous technology set can be measured as:

2 $$D^{t} \left( {x^{t} ,y^{t} } \right) = \inf \left\{ {\theta \left| {\left( {x^{t} ,y^{t} |\theta } \right) \in T_{k}^{t} ,\theta > 0} \right.} \right\},t = 1,2, \ldots ,T$$

where $\theta$ measures the ratio of the actual output to the maximum output in the feasible region, and hence $D^{t} \left( {x^{t} ,y^{t} } \right)$ can represent the technical efficiency of the current assessed DMU compared to the frontier of time t.

The *intertemporal technology set* eliminates the barrier caused by different periods and constructs a reference set for observations of group *k* over the discernible whole time. We denote the intertemporal technology set of observations in group *k* as:

3 $$T_{k} = \left\{ {\left( {x^{t} ,y^{t} } \right)|x^{t} {\text {can produce}} \;y^{t} ,\forall t} \right\}, k = 1,2, \ldots ,K$$

Accordingly, there are *K* separated intertemporal technology sets in the range of all observations. The relevant output distance function on the intertemporal technology set is defined as:

4 $$D^{k} \left( {x^{t} ,y^{t} } \right) = \inf \left\{ {\theta |(x^{t} ,y^{t} |\theta ) \in T_{k} ,\theta > 0} \right\}, k = 1,2, \ldots ,K$$

The *global technology set* is established to envelop all observations all over the investigated time periods and all subgroups, so it can also be deemed as the union of all intertemporal technology sets. Concretely, the global technology set is defined as:

5 $$T^{G} = {\text{conv}}\left\{ {T_{1} \cup T_{2} \cup \ldots \cup T_{K} } \right\}$$

Therefore, the corresponding output distance function on the global technology set is denoted as:

6 $$D^{G} \left( {x^{t} ,y^{t} } \right) = \inf \left\{ {\theta |(x^{t} ,y^{t} |\theta ) \in T^{G} ,\theta > 0} \right\}$$

In order to facilitate the expression of the formulae hereinafter, variables inside the output distance function are expressed in terms of their periods, e.g., $D^{t} \left( t \right)$, $D^{k} \left( t \right)$, and $D^{G} \left( t \right)$. Concretely, we use output-based DEA models under the assumption of variable returns to scale to specify the measurement of three distance functions, which are illustrated in Appendix 1.

Following most previous research, when considering a certain technology set, the output enhancing approaches are defined as follows: (1) for observations located inside the frontier of the contemporaneous technology set, it is useful to reduce the distance between the actual point and the projection point on the frontier, which is formed by technical inefficiency; (2) for observations located on the frontier of a certain contemporaneous technology set, an effective way is to improve the technical merit, so that the height of the belonged frontier is further extended in the direction towards the intertemporal frontier. (3) For the ones situated as the benchmark points on the intertemporal frontier, it is advisable to improve the leading position among groups through improved techniques.

Following similar ideas of output (productivity) improvement decomposition, Oh and Lee (2010) defines the meta-frontier Malmquist productivity index (MMPI) and further decomposes it into three distinct sources, as follows:

7 $$\begin{aligned} M\left( {t,t + 1} \right) & = \frac{{D^{G} \left( {t + 1} \right)}}{{D^{G} \left( t \right)}} \\ & = \frac{{D^{t + 1} \left( {t + 1} \right)}}{{D^{t} \left( t \right)}} \times \frac{{D^{k} \left( {t + 1} \right)/D^{t + 1} \left( {t + 1} \right)}}{{D^{k} \left( t \right)/D^{t} \left( t \right)}} \times \frac{{D^{G} \left( {t + 1} \right)/D^{k} \left( {t + 1} \right)}}{{D^{G} \left( t \right)/D^{k} \left( t \right)}} \\ & = \frac{{{\text{TE}}^{t + 1} }}{{{\text{TE}}^{t} }} \times \frac{{{\text{BPG}}^{t + 1} }}{{{\text{BPG}}^{t} }} \times \frac{{{\text{TGR}}^{t + 1} }}{{{\text{TGR}}^{t} }} \\ & = {\text{EC}} \times {\text{BPC}} \times {\text{TGC}} \\ \end{aligned}$$

where ${\text{TE}}^{l} \left( {l = t,t + 1} \right)$ represents the technical efficiency within a certain period, so EC measures the efficiency change between two adjacent periods. Results of ${\text{EC}} > \left( { = , < } \right)1$ can be considered as the shortening (equivalent, increase) of the relative distance between an observation and its belonged contemporaneous frontier. In addition, ${\text{BPG}}^{l} \left( {l = t,t + 1} \right) > \left( { = , < } \right)1$ signifies the best practice gap between the contemporaneous frontier and its corresponding intertemporal frontier(so that the structural differences between the technologies of production of European vs Chinese universities), and BPC denotes the best practice gap change between two periods. Furthermore, ${\text{BPC}} > \left( { = , < } \right)1$ accounts for the technical progress (stabilization, regress) during the two time. Besides, ${\text{TGR}}^{l} \left( {l = t,\;t + 1} \right)$ stands for the technology gap ratio between the technology within the same group and the global technology, which also reflects the technical leadership of a group of DMUs. Hence, ${\text{TGC}} > \left( { = , < } \right)1$ refers to the advance (constancy, retrogression) in the level of intra-group technology, i.e. how the efficiency of universities vary over time within the two groups.

Following most previous research on higher education efficiency (Abbott & Doucouliagos, 2003; Casu & Thanassoulis, 2006; Ruiz et al., 2015), variable returns to scale assumption (Banker et al., 1984) are employed in the output distance function solution procedure and thereby construct the MMPI hereinafter. We also conduct a test for non-parametric returns to scale, in order to verify the rationality of our hypothesis in Sect. 4.1.

### Variables and data of the assessed universities

Data of the investigated universities for this paper were based on the international university ranking known as playing a crucial role in discussing about the role and position of universities in the world’s higher education. The different ranking systems were made to assess the performance of universities over the world (Anowar et al., 2015; Gnolek et al., 2014). The widely recognized ranking systems—once accepted—are influential to a variety of people, specifically: international students for seeking a right institution for their higher studies; new researchers to carry on scholarly activities and achieve funding facilities; institutions themselves to develop a constructive competition; and also employers to choose appropriate workforce for their business development (Luque-Martínez & Faraoni, 2019; Souto-Otero & Enders, 2017). A review by Anowar et al. (2015) indicated the top four widely accepted ranking systems including THE, ARWU, QS and Webometrics Ranking. Anowar et al (2015, p. 559) showed that “(…) none of these ranking systems can provide satisfactory evaluation in terms of their construct validity and other parameters related to disputation. Overall observation of these four ranking systems reflects the fact that generic challenges include adjustment for institutional size, differences between average and extreme, defining the institutions, measurement of time frame, credit allocation, excellency factors as well as adjustment for scientific fields”. It is observed that since every ranking system has used heterogeneous measures to obtain ranking scores for each university participant, it hardly says which ranking system is more reliable. In addition, Altbach (2015) revealed that teaching quality and assessing the impact of education on students have been not explored in the ranking systems. According to Shattock (2017), the THE ranking has included some teaching related data in terms of a significant weighting to reputation; however, because of this ranking mainly based on research performance, teaching performance seems to be minimally focused.

Despite the fact that the ranking systems are widely criticized for questionable methods and for concepts itself, they have a powerful influence on ranked institutions to perceive who they are as well as to be perceived by others (Altbach, 2015; Shattock, 2017). For example, in a survey of Hazelkorn (2011, p. 86), approximate 70% of institutions want to be in the 10% nationally and in the 25% internationally. Leaders of institutions are often appointed on the ground of a commitment to improve the institution’s ranking (Shattock, 2017). It is clear that no ranking systems are perfect, but they play a crucial guideline for institutions on the way enhancing their academic performance. In a nutshell, while studies on seeking a better method for international university ranking are still on-going, some widely accepted ranking systems, namely THE, ARWU, Webometrics and QS are still used for different purposes in higher education research. On a more institutional ground, they are also commonly employed for identifying those universities that, in specific countries or areas, are considered those elite institutions competing for global positioning.

In such a context, the choice of the group of universities to be included in the study is crucial for the outcome of the productivity analysis of ‘elite’ Chinese and European universities. Several authoritative university rankings in the market provide us with the referable materials for selecting a reasonable sample. Within the scope of three most widely accepted international university rankings, QS places great emphasis on subjective reputation indicators, with weights approaching 50%. ARWU focuses vastly on scientific research indicators, which makes the universities focusing on undergraduate education relatively low rankings. THE uses more indicators in its ranking system than the other two, making its ranking more balanced providing more quantitative information usable for efficiency analyses. Meanwhile, the weight of literature and economic indicators in THE is much higher than that in QS (Olcay & Bulu, 2017). It is clear that THE indicator system is in line with Chinese desire to improve the research quality and treatment of researchers and catch up with the advanced western higher education. Therefore, we employ its records during 2011–2019 to select ‘elite’ Chinese and European universities in this paper. Specifically, annual rankings of universities are employed to determine the average rankings of each listed universities.

After eliminating the universities which have been present in the THE rankings less than seven times during 2011–2019, we choose 50 universities in European Union (EU) countries with the highest average rankings as the EU sample universities. Since the construction and development of Chinese universities started later than the European ones, only seven Chinese universities have been listed in the THE rankings for more than seven times during 2011–2019. However, seven Chinese sample universities are far from adequate in the process of comparing the performance of Chinese and European universities. Therefore, we first searched THE rankings during 2011–2019 and gathered 74 listed Chinese universities, which are regarded as our candidate universities. Therein, several candidate universities are not directly subordinate to the Ministry of Education (MoE) of China, and we cannot collect comparable data of these universities due to the incomplete release of Chinese university data. Ultimately, we choose top 40 samples according to the average rankings from the candidate universities directly under the administration of MoE. These sample universities all belong to Project 211 universities, which are deemed as a group of high-level researching universities in China. Therefore, they can be regarded as representatives of elite universities in China. A detailed list of sample universities is reported in Table 8 (in Appendix 2). Chinese and European universities are naturally divided into two groups because of the obvious differences in educational patterns and evaluation mechanisms between these two groups.

Data about Chinese universities primarily come from Compilation of Science and Technology Statistics of Higher Education Institutions (CSTS-HEI) edited by Chinese Ministry of Education, Graduate Employment Quality Reports (GEQRs) published by sample universities and the InCites database affiliated to Clarivate Analytics.^1^ Data of European universities are mainly extracted from InCites and another database named European Tertiary Education Register (ETER), which is the first comprehensive database registering data of European higher education institutions (HEIs) and has been built through an initiative of the European Commission.

We explore the databases available by the presence of relevant indicators in both sources of data, guided by the selection of inputs and outputs used in the empirical analysis within the existing academic literature. We end up selecting two inputs and three outputs. Table 1 elaborates names, units and data sources of these variables. The selection of these variables refers to the existing literature, considers the availability of data, and takes into account the functions and operational characteristics of colleges and universities. From the perspective of input, both manpower input and capital input are included. In terms of output, the main outputs of both teaching activities and scientific research activities are considered.

**Table 1:** Information about our selected input and output variables

Type	Variables	Units	Data sources
Input	Total current expenditure	Million Euro	EU: ETER
			CN: CSTS-HEI
	Academic staff	Person	EU: ETER
			CN: CSTS-HEI
Output	Students	Person	EU: ETER
			CN: GEQR
	Publication	Piece	Incites database
	Citation	N/A	Incites database

N/A means that there is no unit for the citation variable, which is represented by the category normalized citation impact index; EU and CN represent the belonged groups of samples, i.e., Europe and China

*Total current expenditure* reflects the total cost in research and development (R&D), teaching, and other operational activities of each sample universities. Concretely, “total current expenditure at purchasing power parity” is the exact indicator which we pick from the ETER database. For Chinese sample universities, data of this variable derives from the sum of R&D expenditure, government funding and enterprise funding. Therein, R&D fund refers to the funds for the scientific and educational expenses allocated from the higher authorities, and it is mainly used to carry out the fundamental operational activities. Government fund refers to the research funds provided by government departments to support universities’ participation in scientific research activities. Enterprise fund refers to the research funds obtained from enterprises and institutions outside the university and government departments, and it is also used to support the scientific research activities in universities. To eliminate the influence of inflation and monetary exchange rate across years, we use average exchange rate of RMB against Euro to process the data of “Total current expenditure” of China after gathering three subitems, R&D fund, government fund and enterprise fund.

*Academic staff* refer to the number of staffs (headcounts) engaged in academic activities in each selected university. European data are directly derived from the ETER database, while the Chinese data are arrived from the sum of teaching, researching and R&D personnel. Therein, teaching and research staffs stands for the personnel engaged in teaching and researching activities. R&D staffs refers to personnel engaged in research and development, application of results and services. Unfortunately, there is no detailed explanations on the structure of personnel title, class, etc., so we rely upon simple headcounts.

A methodological, economic note is needed here. The two variables selected as inputs are no doubt correlated, but we decided to include both as a more complete and reliable measure of HEIs’ resources. Indeed, we would like to take explicitly into account that labour intensity (as measured by the number of academic staff) can be different than capital intensity (as measured by expenditures). This is particularly true in cases where the price of labour (i.e. salary) is heterogeneous across institutions. This is certainly the situation we can observe within both Europe and China and across the two areas.

The variable of students refers to the International Standard Classification of Education (ISCED) and denotes it as the *enrolled students at ISCED 6–8*, which corresponds to the total number of students taught or trained in the stages of undergraduate, master and Ph.D. in China. From the year of 2013 on, GEQRs became the main way to reveal the number of students of most Chinese universities. Nevertheless, there is no such uniform publication on the market announcing this figure before 2013. Therefore, data about the number of students consists of two sources. The first part refers to the official enrollment plans released by these universities, which contain data of aggregate enrolments. The second part of the data is collected from GEQRs, which unveil the annual number of graduates of our sample universities. The academic literature in the field discusses whether considering students as inputs (and correspondingly graduates as outputs) or outputs. We opted for the second vision, considering the resources of universities (staff, teaching hours, etc.) are defined on the basis of the number of students, even in cases when they drop out and do not graduate. In this perspective, we consider the number of students a better proxy of the volume of teaching activities actually realized by universities—instead of focusing narrowly on the number of students.

Publications include the *number of papers covered by the Web of Science (WOS) database*, and citation denotes the category *normalized citation impact (CNCI)* of a university. CNCI is a bibliometric indicator which removes the influence of different staff size and subjects’ differences. Data about the bibliometric indicators is collected from the Incites database. This database is one of the products belonging to Clarivate Analytics. To prepare for the retrieval, we set “Retrieval type” as “Organization”, choose “Time period” as “2011–2015”, fill in the name of each university, choose the “Research output” as “document type” (including article and review), choose “Schema” as “ESI” (including SCI and SSCI). Then, we can obtain data of “Web of Science documents” and “Category Normalized Citation Impact^2^” for each university. Therefore, we are sure that data of publication and citation are from the same source and they are comparable at any effect.

The model for representing the productive process of the universities is kept quite simple for allowing the maximum possible comparison between universities in very different contexts. The simplicity of the model allows the generalizability of the production process described and can be used to compare the efficiency of the different organizations (universities) in realizing their base activities, namely teaching and research.

Data of EU samples are collected from the ETER database on April 1, 2019. Until then, the latest data is belonged to the year of 2015. Considering the temporal interval in which both Chinese and European data are available, our investigating period is limited to 2011–2015. Besides, the two different data sources of students in China overlapped in 2013, so that cross-year calculations and comparisons related to Chinese data are all divided into 2011–2013 and 2013–2015 hereinafter. It is worth noting that monetary data of China are converted to Euro by average exchange rate each year from the National Bureau of Statistics of China, as shown in Table 2. To eliminate the inflation factor of Euro in different years, we also employ the average consumer price index (CPI) for EU countries from the OECD database to deal with the data. The CPI data, with 2011 as the base year, are displayed in Table 2.

**Table 2:** Exchange rate and CPI during 2011–2015

Years	Exchange rate of RMB against Euro	CPI
2011	9.0011	1.0000
2012	8.1067	1.0285
2013	8.2219	1.0472
2014	8.1651	1.0603
2015	6.9141	1.0682

**Fig. 1:**
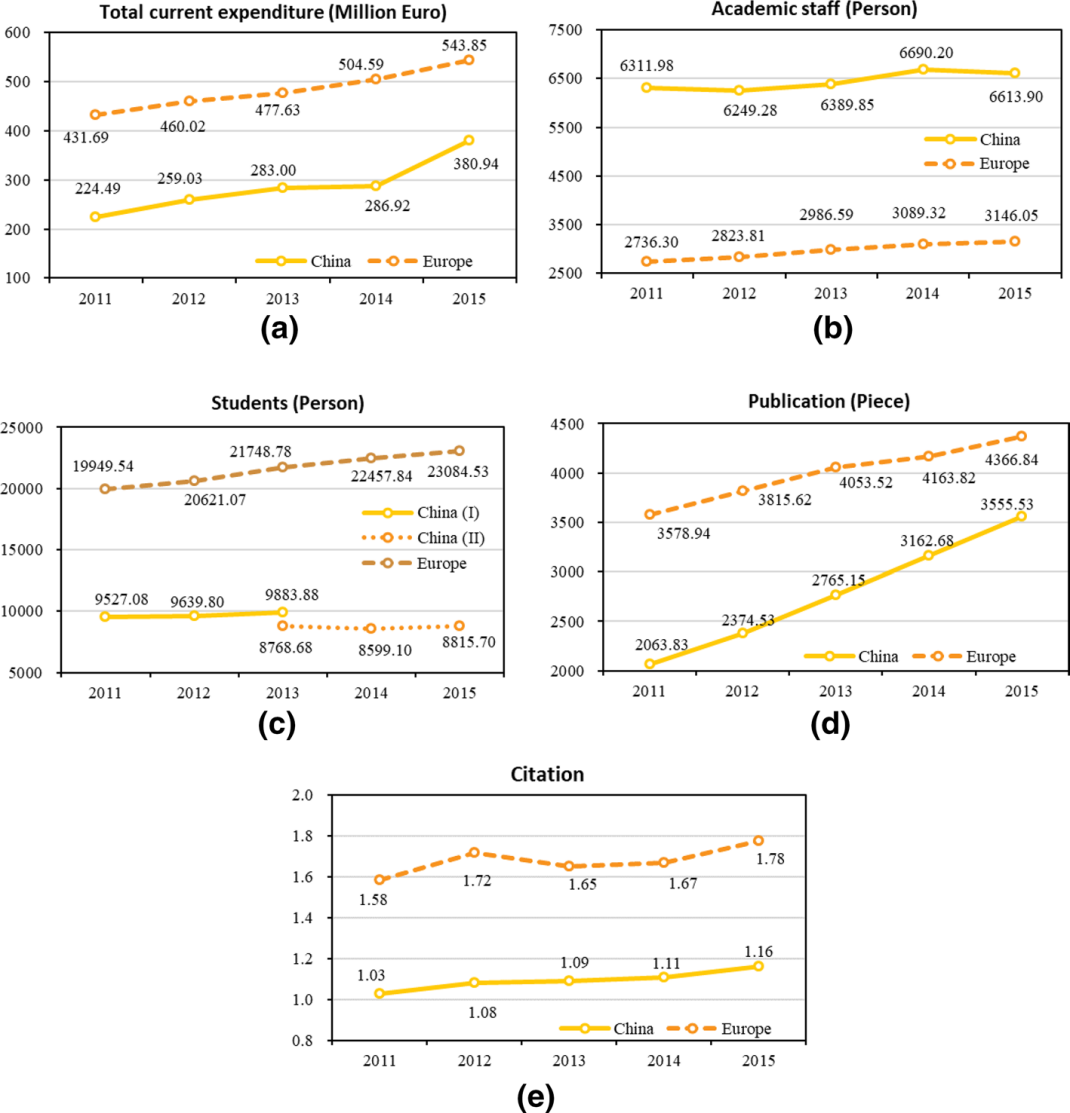
Line charts of variables over the investigated periods

Generally, dissimilar distribution patterns of the original data within different groups can reflect the differences in the operation strategies of universities. Therefore, we provide annual descriptive statistics of Chinese and European universities to make a comparison between the two different groups, which are given in Table 9 (in Appendix 3; moreover, Appendix 3 reports average data by country and year). Furthermore, the annual average values of all variables are used to depict their changing trends, as demonstrated in the line charts of Fig. 1. Peculiarly, we partition the line of students in Fig. 1c to display the different sources of data for the group of Chinese universities.

The changing tendency of variables in Fig. 1 unveils several main differences between Chinese and European elite universities. Firstly, the amount of total current expenditure devoted to European elite universities far exceeds that of Chinese universities, also because of their bigger dimension on average (see number of students later in this section). On the basis of such a large amount of input, they maintain an annual growth rate of expenditures of 6.5%. The annual growth rate in China keeps in the ratio of 17.4%, albeit from a relatively small expenditure base. Secondly, there is a significant difference in the distribution of academic staff between Chinese and European universities, as Chinese universities tend to hire far more academic staff than European ones. Furthermore, academic staff in European universities reveal a general trend of steady rise, while in Chinese universities it remains relatively constant. Thirdly, in terms of the number of papers published and the citation quality, China is attempting to catch up with the European levels at a relatively high speed from a weak and backward basis. When considering publications, both Chinese and European universities are growing fast over the examined period, with average annual growth rates of 18.1% and 5.5%, respectively. However, with regard to the citation quality, Chinese universities keep growing steadily but remain at low levels, while European universities maintain slow growth on a higher level after reaching the secondly high peak in 2012. Due to the interruption of the data sources in China, the exact trend of students trained in Chinese universities is hard to judge, but the number of students within European universities constantly increases at an annual rate of 3.9%.

In general, from the perspective of input and output variable values, top European universities are significantly ahead of top Chinese universities. To identify ways in which the two groups of universities can further improve education performances, we use a meta-frontier approach to derive efficiency and productivity hereinafter, and to allow a fairer and robust comparison of their performance and efficiency.

## Results of the empirical analysis

### Results from the baseline model

To test the return of scale hypothesis satisfied by our data, we employ the non-parametric returns to scale test (Simar & Wilson, 2002; Tran & Dollery, 2021) and give the null assumption that return to scale is constant. We tested data from Chinese and European universities respectively, and both tests rejected the null assumption with *p*-values less than 0.05. Therefore, we employ models based on the assumption of variable returns to scale hereinafter.

Furthermore, since our adopted non-parametric DEA technique is greatly affected by outliers, it is necessary to check whether there are outliers before conducting calculation and analysis (Clermont & Schaefer, 2019). In view of the two different groups of universities in our study, we use the super-efficiency DEA model to calculate the super-efficiency of Chinese universities and European universities, respectively. The results show that only the 33th European university and the 2nd Chinese university present the infeasibility problem in 2011 and 2012. No such problems appear in other universities among other years. Therefore, to include as many universities as possible, we retained all the universities in the following analysis.

For the purpose of seeking out overall performance and differences between the separate groups, the results of meta-frontier productivity and its decompositions are elaborated and presented in detail in the Appendix (Table 11). Although the problem of infeasibility may occur with the VRS-based model, no such cases occur in the calculation process of this paper.

**Fig. 2:**
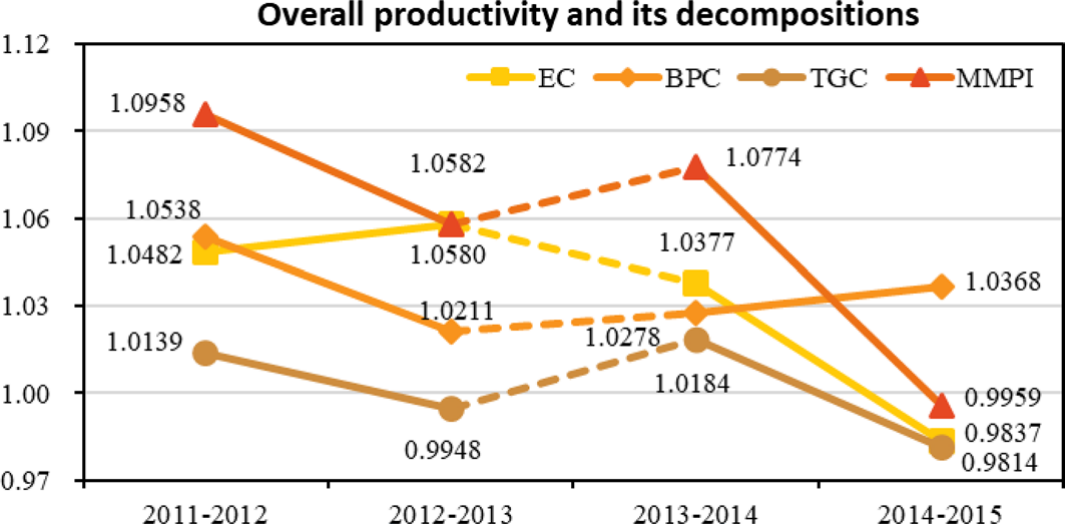
Line chart of the overall productivity and its decomposition

From the view of the whole Chinese and European university system, the MMPI grows with an annual average increase rate of 5.68%. However, values of the productivity index reveal a fluctuant and downward tendency, declining from 1.0958 to 0.9959. It is an indication that the overall productivity growth within China and European higher education system is slowing down. Values of the EC (technical efficiency) share a similar changing pattern with the MMPI, and its declined extent is also dramatic, strikingly descend from 1.0482 to 0.9837. The BPC (technical change over time) is the only decomposed source that stays above unity, and its fluctuation ranges from 1.0211 to 1.0538. Therefore, value of the average BPC ranks highest and acts as the most influential factor shaping the overall performance of the universities under scrutiny. The impact of individual technology leadership, denoted as TGC, keeps fluctuating during this period. It varies slightly on the perimeter of unity, within the range from 0.9814 to 1.0184. Please see detailed computational results in Table 3 and the corresponding line chart in Fig. 2. As anticipated, the overall productivity and its sources are affected by the discontinuity of indicator about students for Chinese universities, thus we use a dotted line to connect the two points 2012–2013 and 2013–2014.

**Table 3:** Overall productivity and its decomposition, all universities together

Periods	EC	BPC	TGC	MMPI
2011–2012	1.0482	1.0538	1.0139	1.0958
2012–2013	1.0580	1.0211	0.9948	1.0582
2013–2014	1.0377	1.0278	1.0184	1.0774
2014–2015	0.9837	1.0368	0.9814	0.9959
Average	1.0319	1.0349	1.0021	1.0568

**Fig. 3:**
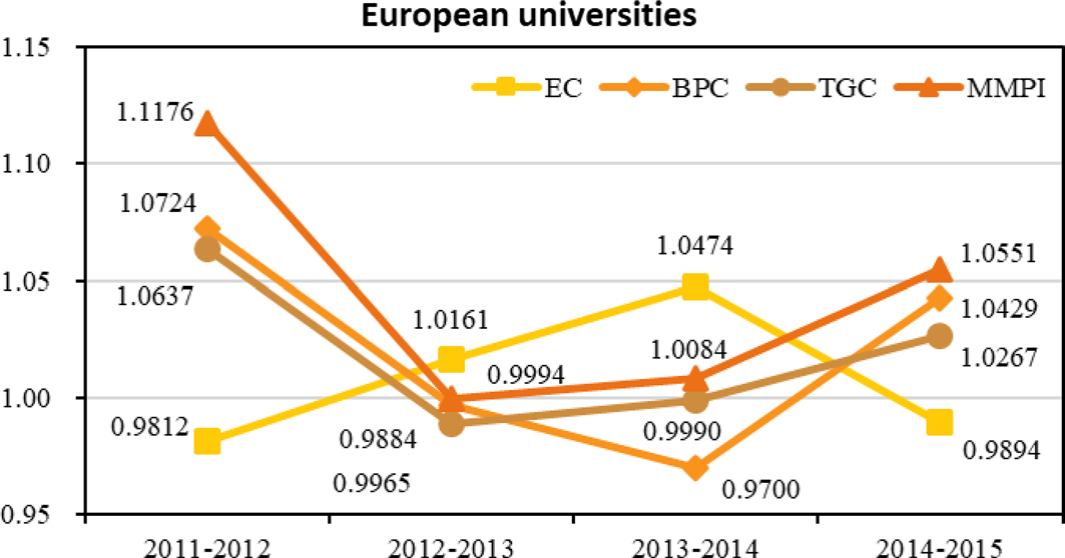
Line chart of the productivity and its decompositions within the European group

**Fig. 4:**
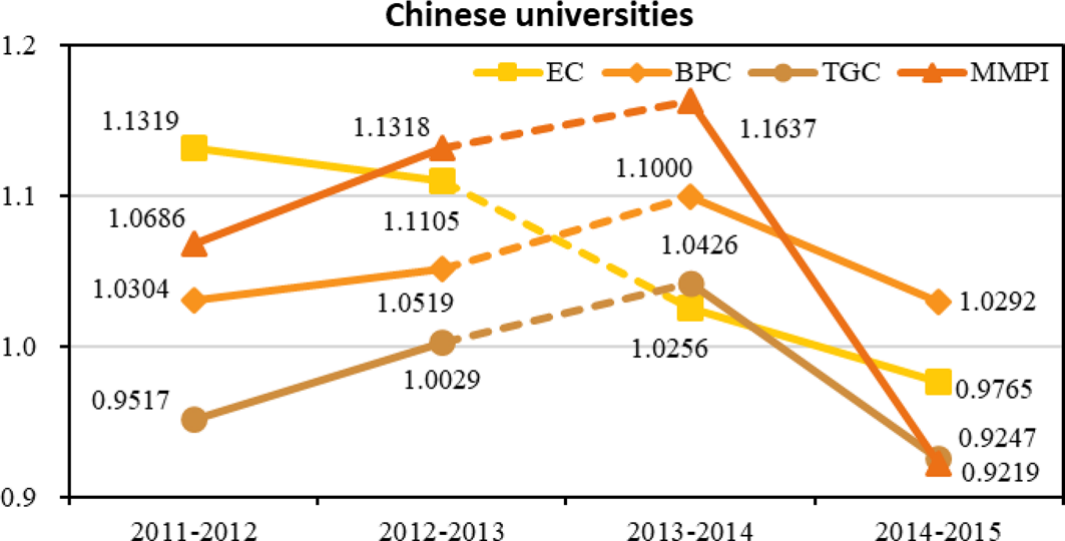
Line chart of the productivity and its decompositions within the Chinese group

On the basis of the assessment of overall condition in Chinese and European tertiary education system, we further conduct an intragroup examination on 50 European sample universities. Numerical results are exhibited in Table 4, and the corresponding trend chart is depicted in Fig. 3. Data sources of the European group is continuous, so Fig. 4 is presented without dotted lines. The MMPI scores in European group experiences the process of falling and then rising with the appearance of a minimum value of 0.9994 at 2012–2013. Averagely, the value of MMPI increases by 4.51% per year, which is inferior to the average of all the samples. From the perspective of sources of MMPI, the EC (technical efficiency) rises at an average rate of 0.85% yearly, while the BPC (technical improvement over time) increased 2.05% annually. EC and BPC show an alternative relationship in the changing process. The TGC (intergroup technology gap) behaves most consistently with the MMPI in this group, with its score falling from 1.0637 to 0.9884 during 2011–2013 and rebounding to 1.0267 until the period 2014–2015.

**Table 4:** Productivity and its decompositions within the European group

Periods	EC	BPC	TGC	MMPI
2011–2012	0.9812	1.0724	1.0637	1.1176
2012–2013	1.0161	0.9965	0.9884	0.9994
2013–2014	1.0474	0.9700	0.9990	1.0084
2014–2015	0.9894	1.0429	1.0267	1.0551
Average	1.0085	1.0205	1.0194	1.0451

When considering the Chinese group, we derive the computational scores and list them in Table 5. The corresponding line chart is demonstrated in Fig. 4 (to be remembered: dotted lines represent the discontinuity in data about students). The average growth value of MMPI reaches 7.15%, a value definitely higher than that reported by European universities. However, the indicator also shows a sharply downward trend to 0.9219 during 2014–2015. Within the decomposed components, EC (technical efficiency) emerges as the key factor with an average annual growth rate of 6.11%, even though it shows a gradually descending tendency. BPC (technical improvement over time) also performs brilliantly, and it increases 5.29% yearly. These figures illustrate that individual efficiency is rising, and the gap between individual universities and group frontier is narrowing. Nevertheless, TGC shows a negative performance, revealing that intra-group technology is somehow inefficient in the period under analysis.

**Table 5:** Productivity and its decompositions within the Chinese group

Periods	EC	BPC	TGC	MMPI
2011–2012	1.1319	1.0304	0.9517	1.0686
2012–2013	1.1105	1.0519	1.0029	1.1318
2013–2014	1.0256	1.1000	1.0426	1.1637
2014–2015	0.9765	1.0292	0.9247	0.9219
Average	1.0611	1.0529	0.9805	1.0715

**Fig. 5:**
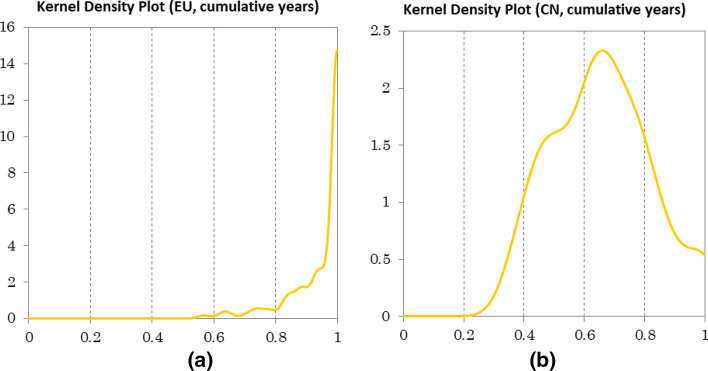
Kernel density plots of TGR (technology gap ratio) in Chinese and European universities

After analyzing the different performance of MMPI and its corresponding decompositions in China and Europe, it is necessary to examine the results of TGR in each group, that is to say the technology gap ratio between the two groups. Even though we have obtained TGC scores, which provide us with the information of leadership changes, we still need TGR to identify which group plays the leading role in technical efficiency during the examined period. Therefore, we examine the TGR results of two groups and depict them in kernel density graphs, as shown in Fig. 5, to recognize differences between groups. TGR scores in EU group reveals centralized distribution in the range of 0.6–1, which indicates that EU universities possess the absolute technology leading position. With regards to the CN group, the concentrated distribution interval of TGR scores has a larger span from 0.2 to 1 and a lower average value. This finding must be interpreted as that there are less internationally leading universities in China compared with in Europe, and a persistent technology gap between the two groups.

### Robustness checks

The results presented in the Sect. 4.1 could be hampered by two main drawbacks of the specification employed for the universities’ production function. On one side, the different quality of publications (i.e. the variable measuring output) can distort the image of efficiency, to the extent to which the quality is higher in the group of Chinese versus European elite universities (or *viceversa*). Given the elite status of the institutions, this eventuality seems remote, but cannot be completely ruled out. On the other side, the differences in salaries for academic staff between Chinese and European Chinese can have an effect on the expenditures (i.e. the input variable), so again distorting the measurement of pure technical efficiency. With these two threats in mind, this section reports the results of two robustness tests that we performed to check the validity of the main findings when different specifications of the production functions are allowed.

Specifically, we re-run all the empirical analyses, modifying the set of variables employed in the efficiency measurement:

- *Robustness test #1* We use two inputs (total current expenditure and academic staff) and three outputs: students, high-quality publications, defined as papers published in Q1 and Q2 journals—as classified by Web of Science—and citations of high-quality publications;
- *Robustness test #2* We use only one inputs (academic staff) and three outputs, as classified in the baseline model (students, publication, citations).

The results of these robustness tests reveal some interesting patterns emerge, which are worthwhile of some discussion.

When considering only high-quality publications (Table 6), the estimated productivity (MMPI) over the period looks lower than that reported through the baseline model (2.11% instead of 5.68%). This is expected, as the number of high-quality publications is (by definition) lower than that of total publications. Also, the productivity increase seems driven by the best practice gap change (BPC) and not by the efficiency variations within groups (EC), the latter being virtually = 1.00. The improvement in the frontier of the two groups is even slightly declining (0.99), a result in line with that emerging from the baseline model. The pattern is very similar across the groups of EU and CN elite institutions, where the only difference is that the productivity increase is a little bit higher for the former (2.39%) than for the latter (1.83%)—but Chinese institutions seem obtaining an improvement of technical efficiency, of about 1.14%.

**Table 6:** Overall productivity and its decomposition, robustness test # 1

Periods	EC	BPC	TGC	MMPI
*Panel A: all universities together*				
2011–2012	0.9625	1.0993	1.0062	1.0635
2012–2013	1.0436	0.9533	0.9994	0.9923
2013–2014	0.9918	1.0117	0.9991	0.9991
2014–2015	1.0011	1.0375	1.0025	1.0407
Average	0.9997	1.0255	1.0018	1.0239
*Panel B: European elite universities*				
2011–2012	0.9625	1.0993	1.0062	1.0635
2012–2013	1.0436	0.9533	0.9994	0.9923
2013–2014	0.9918	1.0117	0.9991	0.9991
2014–2015	1.0011	1.0375	1.0025	1.0407
Average	0.9997	1.0255	1.0018	1.0239
*Panel C: Chinese elite universities*				
2011–2012	1.0457	0.9989	0.9869	1.0300
2012–2013	1.0091	1.0237	0.9884	1.0192
2013–2014	1.0063	1.0116	1.0223	1.0407
2014–2015	0.9847	1.0267	0.9727	0.9831
Average	1.0114	1.0152	0.9926	1.0183

When considering the robustness test #2, where the expenditures are not considered (and all publications and citations are counted, not only high-level ones), again the productivity increase appears somehow lower than the one reported in baseline model (3.51% vs 5.68%), although higher than the one associated with the robustness check #1 (2.11%)—see Table 7. The technical efficiency (EC) and the groups’ specific frontiers (TGC) contribute little—albeit positively—to the total productivity, and the major influence is again exerted by the best practice gap change (BPC). No significant patterns are detected among one of the two groups of elite institutions, which actually behave in a pretty similar manner (see Panels B and C of the Table 7).^3^

**Table 7:** Overall productivity and its decomposition, robustness test # 2

Periods	EC	BPC	TGC	MMPI
*Panel A.: all universities together*				
2011–2012	1.0065	1.0500	1.0077	1.0611
2012–2013	1.0267	0.9820	1.0065	1.0113
2013–2014	0.9935	1.0136	1.0084	1.0151
2014–2015	1.0023	1.0433	1.0068	1.0528
Average	1.0072	1.0222	1.0074	1.0351
*Panel B: European elite universities*				
2011–2012	0.9620	1.1009	1.0085	1.0658
2012–2013	1.0435	0.9433	1.0073	0.9883
2013–2014	0.9979	1.0114	0.9987	1.0074
2014–2015	1.0036	1.0484	1.0000	1.0522
Average	1.0017	1.0260	1.0036	1.0284
*Panel C: Chinese elite universities*				
2011–2012	1.0509	0.9990	1.0070	1.0564
2012–2013	1.0100	1.0207	1.0056	1.0342
2013–2014	0.9892	1.0159	1.0182	1.0229
2014–2015	1.0009	1.0382	1.0135	1.0534
Average	1.0127	1.0184	1.0111	1.0417

Taken together, the results obtained through the two robustness tests proposed here seem confirming the validity of the main findings from the baseline model. Nevertheless, when relaxing some assumptions about the uniformity of the universities’ production functions across groups (namely, comparable quality of all publications and similar prices of inputs), the estimation of productivity increase actually decreases in magnitude. We can then conclude that baseline estimation of productivity growth (5.68%) is an upper bound of the true one, whilst the estimation based on only high-quality publications is a lower bound of it (2.11%).

## Discussion of results and concluding remarks

This paper uses an innovative, meta-frontier Malmquist Productivity Index (MMPI)^4^ to measure productivity efficiency of the elite universities in China and Europe in an overarching framework, considering the period 2011–2015. Answering the research questions of this study, our empirical findings show that universities in the reported sample witnessed a productivity growth of 5.68% on average in the period under analysis. Therefore, this growth fluctuated over the surveyed period and had a decreasing trend. The decomposed components of the productivity index including (1) efficiency change, (2) best practice change and (3) technology gap change illustrated an increase on average at 3.19%, 3.49% and 0.21% respectively. The best practice change demonstrating technical progress among universities during the surveyed period acted as a driven incentive for a rise in overall productivity. These findings suggest that both Chinese and European universities attempted to keep increasing productivity to reach standards of the elite universities. Albeit having started later its investment into projects of building world-class universities, the Chinese HE system’s success can be widely recognised looking at the productivity increase during 2011/12–2014/15. Such a growth in productivity of Chinese universities could not be attainable during the previous post-reform period (1998–2002) as indicated in the work of Ng and Li (2009). However, on the other hand, European elite universities exceed Chinese counterparts in investing in human and financial resources, including the quantity and quality of their academic outputs.

Having a closer look at the performance of European universities under their own frontier, the findings reveal that the average productivity growth of European universities is approximately 4.51%. In comparison with the overall samples, European universities present less technical progress in the productivity change. The initial higher efficiency level can explain why the progress made in the period under analysis can result lower in magnitude. A breakthrough can be seen in the performance of Chinese universities with a productivity growth at 7.15% in which efficiency change and intergroup technology catch-up are key enhancers. This result is slightly lower than the finding found in Song et al. (2019) at 10.3%, who investigated the productivity rise of 58 Chinese universities for the period 2009/10–2015/16. Thus, the study confirms evidence that Chinese universities have been making a brilliant improvement in their academic operations. Also, the increase in productivity points at demonstrating that government policies on Chinese higher education are on a right track to keep supporting and enhancing the higher education projects for the aim of getting Chinese universities into the group of elite universities as soon and many as possible. It may be a role model for Asian nations who would desire to get their tertiary institutions into the world’s high education standards. However, with respect to their individual frontier, Chinese universities still lag behind the current technology leadership conditions. This lag indicates that Chinese universities still have room for improving their performance through a more efficient use of their resources—and specifically, this would be pursued by increasing the research output (publications) more than proportionally when compared with resource investments.

An intriguing question would be investigating the underlying causes for the efficiency and productivity patterns of elite universities, that we synthetized above. Several justifications and acting forces could be explored, including for example (1) the specific effect of targeted policies enacted for elite universities in China, (2) the lack of proper incentives to increase universities in Europe (Aghion et al., 2010) and (3) the potential cost disease effect in the European universities that has been already highlighted in other HE contexts, such as in the USA (Archibald & Feldman, 2008). The (causal) explanation of the phenomena that we have documented is beyond the scope of the present paper. Future research could build on the descriptive findings reported in our paper, following the avenues and hypotheses mentioned above—and additional others.

Meanwhile, the current paper contributes to the literature of higher education by investigating and comparing productivity index of the European and Chinese elite universities, an empirical exercise that has been yet done before. Our empirical findings provide useful information to educational leaders and policy makers to seek appropriate solutions to move nation’s higher education institutions forward. Although European universities have experienced more with the elite university’s standards and got a strong position in the higher education ranking system, they still face some specific challenges (Zhu & Zayim-Kurtay, 2018) as shown in recent studies. For example, European HE institutions suffer a deficiency of interdisciplinary research and lack of national and international collaboration with industry and other tertiary institutions (European Commission, 2005b; European Commission, 2008; LERU, 2006) and other quality variables such as a decline in the number of young people, inequality in low social-economic status students in higher education and inadequate competitive capacity in research and innovation of universities (Eurydice, 2000; European Commission, 2005a; Van Vught, 2011). Following the suggestions by Aghion et al. (2010), further improvements in the productivity could be generated by more universities’ autonomy and extrinsic incentives towards academic excellence (for example, higher shares of competitive-based public funding)—these interventions might be more effective for the group of elite HE institutions, among others. Similar to European universities, Chinese institutions inevitably face challenges on the way to get the world-class university position. That is, the management system should be transparent to enable Chinese universities to achieve their elite and world-class status (Luo, 2013; Ngok & Guo, 2008). In addition, personnel reform and global competition are also influential factors to the elite status of Chinese universities should also be concerned (Song, 2018; Yang, 2000). More generally, the approach of keeping efficiency high by containing costs for inputs (as evidenced in the descriptive statistics in this paper) might be not the proper one when improving performance substantially becomes the key objective. Indeed, the last strategy would imply attracting the best academic talents (i.e. by higher remuneration) and developing top-class facilities and laboratories.

In spite of some clear results presented in the paper, future studies can improve our findings by investigating whether effects of environmental factors on productivity index are affecting the results. In addition, more outputs may be added into the model where appropriate to capture different patterns in production process of universities, especially qualitative outputs as a university’s contribution to the community and outreach (Wolszczak-Derlacz, 2017).

Time dimension is worth to be considered. The analysis presented in this work covers a short period of time, just 4 years—so new studies will be necessary to evaluate the long-run dynamics of efficiency and productivity of elite universities. Moreover, the period considered in this work includes the years during the Great Recession (especially 2011 and 2012). The particular economic conjecture could have affected the production processes of the universities analysed in this work. For example, we are aware that governments and institutions reacted very differently to the specific problem of assuring adequate funding to universities during that period. In the case of China, the employment situation of college graduates turned more severe because of the crisis, and the demand of domestic higher education talent market declined. To address these adverse effects, the government adopted various policy measures. The State invested extra financial input into higher education and established relevant laws and regulations to improve the employment situation. In addition, it also provided additional opportunity to introduce international educational resources and expand multi-regional cooperation and exchanges. In the case of Europe, the European University Association (EUA) reports that some governments (like the French one) invested more resources with a counter-cyclical aim, while others did not—resulting into substantial decline in available resources for universities. These elements call for some further research which looks at these factors more closely, comparing the situation with that consolidated after the financial crisis. From a methodological perspective, although we used a panel structure to track changes in productivity of two groups of universities, a longer span of data would be preferable to provide the productivity index in a more robust manner and thus assess more accurately sustainable development of universities over time.

The results presented in this paper are strongly valid internally but require caution to extrapolate policy implications for non-elite universities, especially poor-performing institutions. Indeed, it might be the case that this latter group is actually more efficient than elite universities—i.e. more able to make the most with available universities. Moreover, universities’ efficiency distributions within Europe and China, as well as across the two areas, might also be different than in the case of the elite HE segment. As a straightforward research direction, future analyses should target a direct comparison of performance and efficiency in the group of second and third tier of HEIs; such an extension would shed more important lights about the overall quality of the HE systems in the two different parts of the globe.

A final note also relates with the radical challenge imposed by the emergence of COVID-19 during 2020. In the different trajectories of evolving performance, Chinese and European universities could have been affected quite heterogeneously by the pandemic, for example because their relative ability of maintaining operations, on-campus lectures, international enrolments, etc. Surely, the investigation of the COVID-19 impact on universities’ efficiency in the international perspective will become a central topic in the empirical HE studies in the coming years.

## Appendix 1

Given that the contemporaneous output distance function reflects the technical efficiency, $D^{l} \left( {x^{t} ,y^{t} } \right)$ can be determined by solving the following output-based radial DEA model:

$$[D^{t} \left( {x_{0}^{t} ,y_{0}^{t} } \right)]^{ - 1} = \max \theta$$

8 $$s.t.\left\{ {\begin{array}{*{20}l} {\mathop \sum \limits_{{j \in T_{k}^{t} }} \lambda_{j} x_{mj}^{t} \le x_{m0}^{t} } \hfill \\ {\mathop \sum \limits_{{j \in T_{k}^{t} }} \lambda_{j} y_{sj}^{t} \le \theta y_{s0}^{t} } \hfill \\ {\mathop \sum \limits_{{j \in T_{k}^{t} }} \lambda_{j} = 1} \hfill \\ {\lambda_{j} \ge 0} \hfill \\ \end{array} } \right.$$

Analogously, the intertemporal output distance function is obtained by solving the following DEA model:

$$[D^{k} \left( {x_{0}^{t} ,y_{0}^{t} } \right)]^{ - 1} = \max \theta$$

9 $$s.t.\left\{ {\begin{array}{*{20}l} {\mathop \sum \limits_{{j \in T_{k} }} \lambda_{j} x_{mj}^{t} \le x_{m0}^{t} } \hfill \\ {\mathop \sum \limits_{{j \in T_{k} }} \lambda_{j} y_{sj}^{t} \le \theta y_{s0}^{t} } \hfill \\ {\mathop \sum \limits_{{j \in T_{k} }} \lambda_{j} = 1} \hfill \\ {\lambda_{j} \ge 0} \hfill \\ \end{array} } \right.$$

In the same way, the global output distance function can be determined based on the following model:

$$[D^{G} \left( {x_{0}^{t} ,y_{0}^{t} } \right)]^{ - 1} = \max \theta$$

10 $$s.t.\left\{ {\begin{array}{*{20}l} {\mathop \sum \limits_{{j \in T^{G} }} \lambda_{j} x_{mj}^{t} \le x_{m0}^{t} } \hfill \\ {\mathop \sum \limits_{{j \in T^{G} }} \lambda_{j} y_{sj}^{t} \le \theta y_{s0}^{t} } \hfill \\ {\mathop \sum \limits_{{j \in T^{G} }} \lambda_{j} = 1} \hfill \\ {\lambda_{j} \ge 0} \hfill \\ \end{array} } \right.$$

## Appendix 2

See Table 8.

**Table 8:** List of universities included in the sample

No.	English Institution name	Country	Group
1	University of Oxford	United Kingdom	EU
2	University of Cambridge	United Kingdom	EU
3	Imperial College London	United Kingdom	EU
4	University College London	United Kingdom	EU
5	London School Economics & Political Science	United Kingdom	EU
6	Karolinska Institutet	Sweden	EU
7	University of Edinburgh	United Kingdom	EU
8	University of Munich	Germany	EU
9	Kings College London	United Kingdom	EU
10	University of Manchester	United Kingdom	EU
11	KU Leuven	Belgium	EU
12	Ruprecht Karls University Heidelberg	Germany	EU
13	University of Bristol	United Kingdom	EU
14	Technical University of Munich	Germany	EU
15	Wageningen University & Research	Netherlands	EU
16	Leiden University	Netherlands	EU
17	Delft University of Technology	Netherlands	EU
18	Utrecht University	Netherlands	EU
19	University of Amsterdam	Netherlands	EU
20	Free University of Berlin	Germany	EU
21	Humboldt University of Berlin	Germany	EU
22	Erasmus University Rotterdam	Netherlands	EU
23	University of Gottingen	Germany	EU
24	Durham University	United Kingdom	EU
25	Lund University	Sweden	EU
26	University of Glasgow	United Kingdom	EU
27	Technical University of Berlin	Germany	EU
28	University of Groningen	Netherlands	EU
29	Uppsala University	Sweden	EU
30	University of Warwick	United Kingdom	EU
31	University of Sheffield	United Kingdom	EU
32	University of York—UK	United Kingdom	EU
33	University of Mannheim	Germany	EU
34	Maastricht University	Netherlands	EU
35	Ghent University	Belgium	EU
36	University of Southampton	United Kingdom	EU
37	Eberhard Karls University of Tubingen	Germany	EU
38	University of St Andrews	United Kingdom	EU
39	University of Freiburg	Germany	EU
40	University of Sussex	United Kingdom	EU
41	Queen Mary University London	United Kingdom	EU
42	RWTH Aachen University	Germany	EU
43	Radboud University Nijmegen	Netherlands	EU
44	Stockholm University	Sweden	EU
45	University of Birmingham	United Kingdom	EU
46	University of Exeter	United Kingdom	EU
47	University of Bonn	Germany	EU
48	Lancaster University	United Kingdom	EU
49	University of Leeds	United Kingdom	EU
50	Eindhoven University of Technology	Netherlands	EU
51	Tsinghua University	Mainland China	CN
52	Peking University	Mainland China	CN
53	Zhejiang University	Mainland China	CN
54	Fudan University	Mainland China	CN
55	Nanjing University	Mainland China	CN
56	Shanghai Jiao Tong University	Mainland China	CN
57	Sun Yat-sen University	Mainland China	CN
58	Wuhan University	Mainland China	CN
59	Huazhong University of Science and Technology	Mainland China	CN
60	Nankai University	Mainland China	CN
61	Tongji University	Mainland China	CN
62	East China normal University	Mainland China	CN
63	Hunan University	Mainland China	CN
64	Renmin University of China	Mainland China	CN
65	Shandong University	Mainland China	CN
66	South China University of Technology	Mainland China	CN
67	Southeast University	Mainland China	CN
68	Tianjin University	Mainland China	CN
69	Xi’an Jiaotong University	Mainland China	CN
70	Xiamen University	Mainland China	CN
71	Central South University	Mainland China	CN
72	China Agricultural University	Mainland China	CN
73	Dalian University of Technology	Mainland China	CN
74	East China University of Science and Technology	Mainland China	CN
75	Huazhong Agricultural University	Mainland China	CN
76	Nanjing Agricultural University	Mainland China	CN
77	Northeast Normal University	Mainland China	CN
78	Sichuan University	Mainland China	CN
79	Beijing Jiaotong University	Mainland China	CN
80	China Pharmaceutical University	Mainland China	CN
81	Chongqing University	Mainland China	CN
82	Jilin University	Mainland China	CN
83	Northeastern University	Mainland China	CN
84	Northwest A&F University	Mainland China	CN
85	Ocean University of China	Mainland China	CN
86	Wuhan University of Technology	Mainland China	CN
87	North China Electric Power University	Mainland China	CN
88	Southwest Jiaotong University	Mainland China	CN
89	Shaanxi normal University	Mainland China	CN
90	University of electronic science and technology of China	Mainland China	CN

## Appendix 3

See Table 9.

**Table 9:** Descriptive statistics of the two groups of universities

Variables (Groups)	Years	Mean	Standard deviation	Min	Max	Median
Total current expenditure (CN)	2011	224.49	164.78	7.97	774.79	188.28
	2012	259.03	190.84	4.33	919.66	242.09
	2013	283.00	209.57	23.18	1033.48	238.99
	2014	286.92	216.32	25.23	1037.70	224.28
	2015	380.94	285.96	23.64	1439.57	323.77
Total current expenditure (EU)	2011	431.69	218.99	92.29	1331.12	418.10
	2012	460.02	236.89	96.13	1440.80	447.12
	2013	477.63	250.79	103.39	1502.73	468.38
	2014	504.59	272.66	106.70	1605.33	481.21
	2015	543.85	300.92	107.85	1793.65	522.15
Academic staff (CN)	2011	6311.98	5289.89	248.00	21,798.00	3795.00
	2012	6249.28	5039.12	412.00	17,879.00	3874.00
	2013	6389.85	5093.03	413.00	18,671.00	4129.50
	2014	6690.20	5300.22	417.00	19,529.00	4194.00
	2015	6613.90	5147.26	439.00	19,013.00	4421.00
Academic staff (EU)	2011	2736.30	1382.10	844.00	5811.00	2444.16
	2012	2823.81	1433.89	875.00	5998.90	2487.60
	2013	2986.59	1555.92	956.00	6979.83	2602.88
	2014	3089.32	1616.20	1024.00	7083.84	2690.00
	2015	3146.05	1634.96	973.00	7085.86	2750.00
Students (CN)	2011	9527.08	3304.53	3818.00	17,715.00	8305.50
	2012	9639.80	3194.05	3835.00	17,875.00	8422.50
	2013 (I)	9883.88	3187.43	3929.00	17,955.00	8796.00
	2013 (II)	8768.68	3013.51	3996.00	17,569.00	8102.50
	2014	8599.10	2916.12	4160.00	16,520.00	7827.50
	2015	8815.70	2866.19	4193.00	16,972.00	8023.50
Students (EU)	2011	19,949.54	8017.39	7753.35	40,408.00	19,493.38
	2012	20,621.07	8006.03	8266.71	42,849.00	20,132.00
	2013	21,748.78	9047.02	8125.00	50,114.00	21,726.50
	2014	22,457.84	9166.42	8418.00	50,446.00	22,069.50
	2015	23,084.53	9070.06	8482.00	50,382.00	23,320.00
Publication (CN)	2011	2063.83	1452.30	419.00	5903.00	1679.00
	2012	2374.53	1656.95	500.00	6604.00	1983.00
	2013	2765.15	1931.81	613.00	7572.00	2201.00
	2014	3162.68	2121.97	726.00	8503.00	2512.50
	2015	3555.53	2349.29	800.00	9400.00	2724.00
Publication (EU)	2011	3578.94	1842.31	354.00	8931.00	3380.50
	2012	3815.62	1965.67	356.00	9389.00	3539.50
	2013	4053.52	2117.67	384.00	10,494.00	3897.00
	2014	4163.82	2144.11	373.00	10,747.00	3922.00
	2015	4366.84	2282.40	439.00	11,496.00	4203.00
Citation (CN)	2011	1.03	0.17	0.74	1.51	1.05
	2012	1.08	0.16	0.79	1.35	1.10
	2013	1.09	0.14	0.83	1.43	1.10
	2014	1.11	0.12	0.85	1.39	1.12
	2015	1.16	0.15	0.92	1.45	1.13
Citation (EU)	2011	1.58	0.16	1.21	1.99	1.57
	2012	1.72	0.20	1.39	2.35	1.66
	2013	1.65	0.16	1.22	2.00	1.67
	2014	1.67	0.19	1.25	2.14	1.67
	2015	1.78	0.22	1.17	2.15	1.81

Data sources of indicator students are divided into two parts, which overlap in the year of 2013, so descriptive statistics corresponding to the two data sources are both provided here. For the convenience of distinction, I and II are employed in parentheses to identify the data sources

## Appendix 4

See Table 10.

**Table 10:** Descriptive statistics, by group (CN and EU) and year

		Students	Current expenditures	Current expenditures/students	Academic staff	Academicstaff/students
Chinese	2011	9527.08	224,486,781.00	24,259.61	6311.98	0.61
European	2011	19,949.54	431,689,717.36	23,154.54	2736.30	0.15
Chinese	2012	9639.80	251,847,719.94	26,190.82	6249.28	0.60
European	2012	20,621.07	447,269,999.73	23,231.82	2823.81	0.15
Chinese	2013	9883.88	270,244,603.61	27,680.99	6389.85	0.61
Chinese *	2013*	8,768.68	270,244,603.61	31,704.58	6389.85	0.69
European	2013	21,748.78	456,100,809.95	22,916.67	2986.59	0.15
Chinese	2014	8599.10	270,603,923.73	32,617.15	6690.20	0.74
European	2014	22,457.84	475,891,766.76	23,031.29	3089.32	0.15
Chinese	2015	8815.70	356,622,145.58	41,254.35	6,613.90	0.70
European	2015	23,084.53	509,127,793.36	23,952.02	3146.05	0.14

*Here, we add this line because data sources of student in Chinese universities contains two parts. This is explained in the paper (section about Data)

## Appendix 5

See Table 11.

**Table 11:** Meta-frontier productivity and its decompositions of the two groups of universities

DMU	2011–2012				2012–2013				2013–2014				2014–2015			
	EC	BPC	TGC	MMPI	EC	BPC	TGC	MMPI	EC	BPC	TGC	MMPI	EC	BPC	TGC	MMPI
1	1.0000	1.0724	1.0191	1.0929	1.0000	1.0301	0.9708	1.0000	1.0000	1.0081	1.0000	1.0081	1.0000	1.0000	1.0000	1.0000
2	0.9970	1.1108	1.0307	1.1415	0.8669	1.0924	0.9619	0.9110	1.0779	0.9094	1.0000	0.9802	0.9655	1.1010	1.0000	1.0629
3	0.9493	1.0572	1.0034	1.0070	1.0067	1.0407	0.9888	1.0358	1.1050	0.9441	1.0011	1.0443	1.0000	1.0893	0.9989	1.0881
4	1.0000	1.0000	1.0000	1.0000	1.0000	1.0537	0.9948	1.0482	1.0000	0.9660	1.0000	0.9660	1.0000	1.0352	1.0000	1.0352
5	1.0846	1.0000	1.7903	1.9417	1.0000	0.9090	0.8383	0.7620	0.9850	0.9572	0.8436	0.7954	1.0030	1.0144	1.1452	1.1652
6	0.9545	1.0411	1.0429	1.0364	1.1167	1.0082	1.0087	1.1356	1.0661	0.9516	1.0066	1.0212	1.0000	1.1013	0.9934	1.0941
7	1.5674	1.1706	0.9545	1.7513	0.6530	0.8178	1.0768	0.5750	1.5314	0.7521	1.0000	1.1517	0.6400	1.6260	1.0000	1.0407
8	0.9352	1.3997	1.0818	1.4161	1.0992	1.0196	0.9995	1.1202	1.1948	0.9984	1.0000	1.1929	0.9029	1.0986	1.0000	0.9919
9	0.8355	1.2985	1.0519	1.1412	1.1354	0.9481	1.0224	1.1007	1.0610	1.0643	1.0000	1.1292	0.9503	1.0899	1.0000	1.0358
10	0.8600	1.0599	1.0004	0.9119	0.8628	1.2072	1.0119	1.0540	0.9016	1.0697	1.0000	0.9645	1.1540	0.9796	1.0000	1.1304
11	1.0000	1.0244	1.0075	1.0321	1.0000	1.4025	0.9648	1.3532	1.0000	1.0000	1.0000	1.0000	1.0000	1.0000	1.0000	1.0000
12	1.3228	0.8986	1.3145	1.5625	1.0000	1.0844	0.9222	1.0000	1.0000	1.0197	0.9955	1.0151	1.0000	1.1390	1.0057	1.1455
13	0.9595	1.1310	1.1429	1.2403	1.1907	0.7580	0.9371	0.8458	0.7769	1.2209	0.9691	0.9191	1.0663	1.1257	1.0206	1.2250
14	1.0174	1.0516	1.0182	1.0893	1.0366	1.0705	1.0464	1.1612	1.3059	0.8468	1.0000	1.1059	1.0685	1.0494	1.0000	1.1213
15	0.8542	1.1693	0.9751	0.9739	1.1256	0.9389	1.0317	1.0903	1.0672	0.9797	0.9776	1.0221	1.0152	1.0400	1.1383	1.2019
16	0.8520	1.2601	1.0223	1.0976	0.9577	0.9989	1.0013	0.9579	1.0098	0.9741	1.0075	0.9910	0.9867	1.0343	1.0103	1.0310
17	1.0055	1.0373	1.0189	1.0628	1.0055	0.9871	1.0186	1.0109	1.0401	0.9724	1.0184	1.0299	1.0088	1.0154	1.0249	1.0498
18	1.0352	1.0770	0.9962	1.1107	1.0000	1.0627	0.9772	1.0384	1.0000	0.9880	1.0000	0.9880	0.9780	1.0090	1.0000	0.9868
19	1.0000	1.0383	0.9940	1.0320	1.0000	0.9969	1.0031	1.0000	1.0000	1.0267	0.9908	1.0173	1.0000	1.0000	1.0000	1.0000
20	1.0269	1.0325	1.0386	1.1013	0.9391	1.0401	1.0036	0.9803	1.0983	0.9722	1.0000	1.0678	1.0051	1.0658	1.0000	1.0713
21	1.0000	1.0020	1.0204	1.0225	1.0000	1.0000	1.0000	1.0000	1.0000	0.9290	1.0000	0.9290	1.0000	1.0764	1.0000	1.0764
22	1.0000	0.9919	1.0668	1.0582	1.0000	1.0051	0.9949	1.0000	1.0000	0.9990	0.9838	0.9828	1.0000	1.0111	1.0196	1.0309
23	0.9519	1.0712	1.2265	1.2506	1.0243	1.0215	0.8463	0.8855	1.3510	0.7825	1.0822	1.1441	1.1231	1.1623	1.0862	1.4179
24	0.9156	1.0685	0.9057	0.8861	1.0215	0.9662	1.0886	1.0745	1.0222	0.9744	0.9430	0.9393	0.9262	1.0324	1.0425	0.9968
25	0.9066	1.0830	1.0095	0.9912	0.9608	1.0231	1.0007	0.9837	1.0272	0.9996	1.0000	1.0267	0.9853	1.0149	1.0000	1.0000
26	0.8644	1.1165	1.0244	0.9886	1.0785	0.8966	1.0240	0.9901	0.8959	1.0744	0.9620	0.9260	0.9459	1.0179	1.0202	0.9822
27	0.9929	1.0226	1.0527	1.0690	0.9911	1.0511	1.0108	1.0530	1.0826	0.9590	1.0171	1.0560	1.1459	0.9140	1.0279	1.0766
28	0.9461	1.0955	1.0123	1.0492	0.9976	1.0137	1.0007	1.0120	1.0226	1.0132	1.0000	1.0361	1.0512	0.9844	1.0116	1.0468
29	0.8260	1.1614	1.0098	0.9688	1.0344	0.9936	1.0286	1.0571	0.9958	0.9799	1.0000	0.9759	1.0250	0.9912	1.0244	1.0408
30	0.8872	1.0796	1.0069	0.9644	0.9662	0.9823	1.0407	0.9877	0.8950	0.9763	0.9813	0.8574	0.9765	1.0223	1.0236	1.0219
31	0.8920	1.0607	1.0068	0.9525	1.0016	0.9967	1.0142	1.0125	0.9886	0.9556	1.0007	0.9453	0.9421	1.0218	1.0116	0.9739
32	0.7650	1.1698	0.8155	0.7298	1.0614	0.9281	1.1362	1.1193	0.9458	1.0153	0.8629	0.8286	1.0313	1.0306	1.2608	1.3399
33	1.0000	1.0000	0.9250	0.9250	1.0000	0.9600	0.8930	0.8573	1.0000	1.0073	1.0433	1.0509	1.0000	1.0341	1.0650	1.1013
34	1.0526	1.0317	1.1253	1.2220	1.0270	0.9697	0.8903	0.8866	1.0828	0.9564	0.9883	1.0235	0.9636	1.0227	1.0861	1.0703
35	1.0000	1.0773	1.0096	1.0876	1.0000	1.1962	0.9905	1.1848	1.0000	0.9990	1.0000	0.9990	1.0000	1.0010	1.0000	1.0010
36	0.9279	1.0532	1.0143	0.9912	1.0372	0.9817	1.0341	1.0530	1.0265	0.9281	1.0283	0.9797	0.9696	1.0649	1.0204	1.0536
37	1.0029	1.0089	1.0884	1.1014	1.1584	0.8887	1.1327	1.1661	1.1848	0.7946	0.9064	0.8534	0.9081	1.0176	1.0591	0.9787
38	1.0031	0.9775	1.2560	1.2315	1.0246	0.9607	0.9086	0.8943	1.0000	1.0000	0.9913	0.9913	1.0000	0.9925	1.0815	1.0734
39	0.9858	1.0071	1.0094	1.0021	1.1625	0.8805	1.0570	1.0819	1.1646	0.8192	0.9291	0.8864	0.9493	1.0143	0.9464	0.9112
40	1.0000	1.0000	1.2557	1.2557	0.9840	0.9299	0.7858	0.7190	1.0163	1.0754	1.1837	1.2937	1.0000	0.9660	1.0352	1.0000
41	1.1885	1.0081	1.2946	1.5512	0.8985	0.9375	0.8467	0.7132	1.0169	0.9863	1.0879	1.0912	1.0000	1.0288	1.0261	1.0557
42	1.0482	1.0532	1.0394	1.1474	1.0088	1.0730	1.0379	1.1234	1.0998	0.8915	1.0154	0.9956	1.1144	0.9590	1.0067	1.0759
43	0.9431	1.0340	1.0202	0.9948	1.0138	0.9989	1.0301	1.0433	1.1390	0.8957	1.0026	1.0228	0.9052	1.1020	1.0050	1.0025
44	1.0000	1.1325	1.0244	1.1601	1.0000	1.0000	1.0000	1.0000	1.0000	1.0000	1.0000	1.0000	1.0000	0.9360	1.0000	0.9360
45	0.9041	1.0980	1.0162	1.0088	1.0774	0.9906	1.0271	1.0962	0.9920	0.9500	1.0000	0.9425	1.0241	1.0204	1.0223	1.0683
46	0.8400	1.1097	1.0120	0.9433	1.0835	0.9067	1.0703	1.0515	0.9478	1.0523	0.9630	0.9605	0.9522	1.0287	1.0405	1.0192
47	1.0594	1.0890	1.1505	1.3273	1.1644	0.8981	0.9918	1.0372	1.2359	0.9599	1.0072	1.1948	0.9852	1.0166	0.9953	0.9969
48	1.0030	0.9840	1.1351	1.1202	0.9770	0.9474	0.8426	0.7799	0.9980	0.9822	1.1541	1.1312	0.8738	1.0235	0.9645	0.8627
49	0.8101	1.1237	0.9922	0.9032	1.0098	1.0002	1.0137	1.0238	0.9984	0.9718	0.9960	0.9664	0.9659	1.0018	1.0101	0.9774
50	1.0885	0.9809	1.1544	1.2326	1.0431	0.9603	0.9018	0.9033	1.0214	0.9520	1.0100	0.9821	0.9641	1.0217	1.1051	1.0885
51	1.0000	1.3845	1.3017	1.8022	1.0000	1.2180	1.1568	1.4090	1.0000	0.9770	1.0568	1.0324	1.0000	1.0235	0.9693	0.9921
52	1.0000	1.0000	1.0000	1.0000	1.0000	1.0000	0.8370	0.8370	1.0000	1.0000	1.0000	1.0000	1.0000	1.0000	1.0000	1.0000
53	1.0000	1.3468	0.8584	1.1561	1.0000	1.1976	1.1814	1.4148	1.0000	1.2546	0.9616	1.2065	1.0000	1.1338	0.8430	0.9558
54	1.2030	0.9493	0.9396	1.0730	1.2963	1.2551	0.8781	1.4286	1.2222	1.1117	0.9208	1.2512	1.0727	1.0081	0.9900	1.0706
55	0.9448	1.0376	0.9525	0.9337	1.1606	1.1004	0.9699	1.2387	1.5943	1.1186	1.0553	1.8821	0.9803	0.9416	0.9151	0.8447
56	1.0100	1.0828	1.0764	1.1772	1.1610	0.8761	0.9761	0.9928	0.8459	1.2195	1.0075	1.0393	0.8401	1.1624	1.0171	0.9931
57	1.2009	1.0867	0.8530	1.1132	1.5856	0.7891	0.8941	1.1186	1.0947	0.8882	1.0665	1.0369	0.9515	1.0760	0.8136	0.8329
58	1.2987	0.9943	0.8645	1.1164	1.0000	1.1223	1.2027	1.3498	1.0000	1.0000	0.8484	0.8484	1.0000	1.0000	1.1144	1.1144
59	1.2500	1.0776	0.9219	1.2418	1.2227	0.9902	0.8661	1.0487	1.1118	1.0918	0.9673	1.1743	1.0930	1.1014	0.9364	1.1272
60	1.1002	0.9548	0.9696	1.0185	1.1479	1.0474	0.8037	0.9663	0.8757	1.0832	1.0432	0.9896	1.0284	0.8949	0.9948	0.9156
61	1.0000	0.9240	0.8855	0.8182	1.0000	1.0823	1.0267	1.1111	1.0000	0.9370	1.1017	1.0323	1.0000	1.0672	0.9297	0.9922
62	0.8260	0.8802	0.9835	0.7151	1.2107	0.7826	0.9543	0.9041	0.7190	1.1447	1.0412	0.8569	0.7719	1.2258	0.8272	0.7828
63	0.8230	1.1961	0.9674	0.9523	1.0389	1.1859	1.0428	1.2847	1.1392	1.1154	1.1156	1.4176	0.8101	0.9817	0.9797	0.7791
64	1.1061	0.9742	1.0009	1.0785	1.0647	1.0895	0.9039	1.0485	1.0516	1.1022	1.1048	1.2805	1.0143	0.9777	0.9300	0.9223
65	1.0000	1.1620	1.1961	1.3898	1.0000	1.2788	0.9620	1.2302	1.0000	1.2277	1.0741	1.3187	1.0000	1.0246	1.0167	1.0417
66	0.8456	0.9845	0.9327	0.7765	0.9884	1.0650	0.9884	1.0404	1.0762	1.0690	0.9989	1.1492	0.9578	0.9501	0.9496	0.8641
67	0.9624	1.0101	0.9085	0.8832	1.0593	1.0733	1.0170	1.1563	1.1503	1.0565	1.0103	1.2277	0.9311	0.9630	0.9153	0.8207
68	1.0000	0.9620	0.9574	0.9210	1.0000	1.0395	0.9917	1.0309	1.0000	1.0417	0.9869	1.0281	1.0000	1.0000	1.0417	1.0417
69	1.1808	0.9823	0.9257	1.0737	1.2632	0.9167	1.0082	1.1674	0.7704	1.2136	1.2522	1.1708	1.0878	0.8949	0.8899	0.8663
70	3.6815	0.6039	1.0196	2.2667	0.6002	1.2801	0.9500	0.7299	1.0000	1.3132	0.9226	1.2115	0.8639	1.0728	0.8878	0.8228
71	2.0476	0.9709	0.8542	1.6982	0.9499	0.9804	0.9826	0.9151	1.0282	1.0413	0.9846	1.0542	0.8590	1.1719	0.8690	0.8747
72	1.0000	1.0996	1.0124	1.1133	1.0000	1.1765	1.1274	1.3263	1.0000	1.2920	0.8571	1.1074	1.0000	1.0000	0.8659	0.8659
73	1.0000	1.0000	0.9945	0.9945	1.0000	0.9190	0.9975	0.9167	0.8740	1.0746	1.1098	1.0424	0.8593	0.9989	0.9432	0.8096
74	1.0000	1.0000	0.7615	0.7615	1.0000	1.0000	1.1227	1.1227	0.9978	1.1046	0.9893	1.0903	1.0905	0.9740	1.0821	1.1494
75	1.0256	1.1481	0.9695	1.1415	0.9806	1.0375	0.9504	0.9669	1.3107	1.1313	0.9674	1.4346	0.8664	0.9943	0.9219	0.7941
76	1.0000	0.9290	1.0051	0.9338	0.9850	0.9977	1.0199	1.0024	0.8560	1.0190	1.3662	1.1917	1.1933	1.0060	0.7265	0.8721
77	1.0331	1.1757	0.8446	1.0258	1.0000	1.0905	1.1419	1.2453	1.0000	1.0215	1.1870	1.2125	1.0000	1.0000	1.0495	1.0495
78	1.0000	0.9680	0.5739	0.5556	1.0000	1.0331	0.9164	0.9467	1.0000	1.0000	0.9818	0.9818	1.0000	0.9640	0.8933	0.8611
79	1.0950	1.0341	0.9915	1.1227	1.7494	0.5949	0.9569	0.9960	0.8306	1.4756	0.9175	1.1245	0.7065	1.6566	0.9609	1.1246
80	1.1235	0.8882	0.9298	0.9279	1.4859	0.9280	0.9281	1.2798	1.0000	1.2729	1.0559	1.3440	1.0000	1.1403	0.9303	1.0607
81	1.2003	0.9054	0.9435	1.0254	1.3324	1.0165	1.1420	1.5467	1.0705	1.0889	1.0160	1.1843	1.0132	1.0020	0.8982	0.9119
82	1.0000	1.1881	0.9811	1.1657	1.0000	1.0695	1.2542	1.3414	1.0000	1.0417	1.2323	1.2837	1.0000	1.0000	0.7640	0.7640
83	1.4851	0.7413	0.8137	0.8958	1.0050	1.2621	0.7945	1.0078	0.8200	1.3645	1.3917	1.5572	1.1061	0.9414	0.7624	0.7938
84	1.0000	0.9683	0.9642	0.9337	1.0000	1.3624	1.0444	1.4228	1.0000	1.0000	1.0251	1.0251	1.0000	1.0000	0.9889	0.9889
85	1.0000	1.0399	0.9386	0.9761	0.7680	1.0661	1.1076	0.9069	1.1136	1.0965	1.0715	1.3083	1.0000	1.0000	1.0984	1.0984
86	1.0909	0.8475	0.9620	0.8894	0.4049	1.1668	1.4037	0.6632	1.1252	0.9116	0.9936	1.0192	1.0736	0.8712	0.8956	0.8377
87	1.1281	1.0261	1.0448	1.2094	1.6949	1.1153	0.6692	1.2650	1.0168	1.0610	0.9874	1.0651	1.0183	0.9747	0.8689	0.8624
88	1.0442	0.9296	0.9515	0.9236	1.2269	1.1720	1.0009	1.4393	1.0826	1.0915	1.0477	1.2381	0.9362	0.8978	0.8270	0.6952
89	1.1391	1.0414	1.0049	1.1921	1.3459	1.1005	1.0335	1.5308	0.9525	1.1011	0.9829	1.0308	1.0478	0.9797	0.9106	0.9348
90	0.4320	1.7218	1.0109	0.7519	1.6906	0.5987	0.9097	0.9208	1.2932	0.8463	1.0033	1.0980	0.8854	1.0958	0.7715	0.7485

## Footnotes

1 https://incites.thomsonreuters.com/#/analytics.

2 CNCI of a document is calculated by dividing the actual count of citing items by the expected citation rate for documents with the same document type, year of publication and subject area. CNCI of a set of documents, for example the collected works of a university in this paper, is the average of the CNCI values for all the documents in the set. More detailed information can be seen at: https://incites-help-clarivate-com-s.webvpn.las.ac.cn/Content/Indicators-Handbook/ih-normalized-indicators.htm#.

3 It must be noted here that the use of DEA for determining efficiency is somehow responsible for the effect described here. Indeed, as the number of inputs is reduced, the algorithm for calculating efficiency has less degrees of freedom to identify optimal virtual weights that maximize efficiency itself—of course, under the assumption that heterogeneity in the use of inputs for producing outputs, across institutions. Nevertheless, the main aim for this robustness test is to check the qualitative similarity of results, and not their quantitative homogeneity.

4 The academic literature is currently debating the validity of specific extensions of the traditional Malmquist Index—see, for example, Afsharian et al. (2018). This paper is not entering the methodological controversy, and we decided to use the mostly adopted approach in some recent literature about the measurement of productivity change. Nonetheless, future research must be devoted to test the validity of results when adopting alternative methodologies under different assumptions and specifications of the productivity change.
